# Neuropsychiatric consequences of COVID-19 related olfactory dysfunction: could non-olfactory cortical-bound inputs from damaged olfactory bulb also contribute to cognitive impairment?

**DOI:** 10.3389/fnins.2023.1164042

**Published:** 2023-06-22

**Authors:** Bernat Kocsis, Benjamin Pittman-Polletta

**Affiliations:** ^1^Department of Psychiatry, Harvard Medical School, Beth Israel Deaconess Medical Center, Boston, MA, United States; ^2^Department of Mathematics and Statistics, Boston University, Boston, MA, United States

**Keywords:** respiratory-related coupling, mechanoceptive OB input, cortical oscillations, brain network activity, rhythmic neural synchronization

## Introduction

Disturbances in smell emerged at the very beginning of the pandemic as the predominant neurological symptom of COVID-19 (Boscolo-Rizzo et al., 2020; Menni et al., 2020) providing evidence of COVID-19 related neurological abnormalities originating from pathology of the olfactory epithelium. The very first prospective imaging studies (MRI scans 3–4 months after COVID-19 hospitalization in Wuhan) reported significant changes in gray matter volume correlated with loss of smell and memory loss, primarily found in cingulate gyrus, piriform cortex, and hippocampus (Lu et al., 2020). Since then, the available data have substantially expanded and the focus shifted to long-term sequelae in which cognitive and mental functioning are prominently featured in post-COVID and long-COVID conditions [see rev. (Rogers et al., 2020; Xydakis et al., 2021; Batiha et al., 2022; Doty, 2022; Hasegawa et al., 2022; Kay, 2022; Lippi et al., 2023)]. Research in this area is rapidly progressing; the most recent articles are being collected in this Special Issue underlining that investigation of the neuropsychiatric sequelae of olfactory dysfunction related to COVID-19 infection is particularly critical to characterize the pathological effects of COVID-19 on brain function and to develop strategies to improve patient's quality of life and mental wellbeing.

In this paper we call attention to potential benefits these studies may gain from a wider approach, including respiratory related oscillations (RRO) in forebrain structures induced by rhythmic nasal airflow. It may help in two major aspects of this research, concerning the two “ends” of the pathology of the central olfactory processing networks, extending from the olfactory bulb (OB) all the way to cortical networks (Xydakis et al., 2021). These are two points where processing of distinct sensory inputs from the OB significantly overlaps and where investigating RRO mechanisms may help to understand (1) how smell loss is caused by SARS-2-COV infection which does not directly attack olfactory sensory neurons (OSN) (Cooper et al., 2020; Iadecola et al., 2020; Doty, 2022; Las Casas Lima et al., 2022; Rodriguez-Sevilla et al., 2022; Butowt et al., 2023) and (2) how olfactory dysfunction advances to a complex condition of diverse cognitive and emotional disturbances (Putri et al., 2021; Soltani et al., 2021; Vanderlind et al., 2021; Batiha et al., 2022; Kay, 2022; Crook et al., 2023).

Respiratory rhythmic modulation of a wide range of cognitive functions has been reported both in rodents and human, from sensory processing and motor coordination to various memory functions [rev. (Heck et al., 2019)]—i.e. not directly related to gas exchange. In rodents, during exploration associated with sniffing, respiratory rate accelerates to match the frequency of hippocampal (HPC) theta rhythm, an intrinsic brain oscillation. During these episodes, RRO play a key role in synchronizing sensory sampling in OB on one hand and rhythmic fluctuations in excitability of neurons involved in central processing in HPC and piriform cortex, on the other. Outside of sniffing episodes, when respiration is in the delta range, OB RRO synchronize instead with frontal cortical delta oscillations. In rats and mice, these waking delta oscillations include task-related intrinsic oscillations (Fujisawa and Buzsaki, 2011; Dejean et al., 2016; Karalis et al., 2016; Furtunato et al., 2020) and are markedly different from the broad-band thalamo-cortical delta rhythms of deep sleep (Pittman-Polletta et al., 2018), being spectrally narrow-band, cortically generated, hierarchically nested with gamma oscillations (Hunt et al., 2017; Pittman-Polletta et al., 2018), and associated with various cognitive functions (Nacher et al., 2013; Hall et al., 2014; Riecke et al., 2015; Hunt et al., 2017). Since respiration is slower in humans than rodents, whereas the frequencies of brain rhythms are evolutionary wellpreserved (Buzsaki and Draguhn, 2004), RRO in humans exhibit a different form of coupling with forebrain oscillations. It periodically modulates the levels of oscillatory activity in forebrain circuits, including both “slow” delta and theta rhythms as well as “fast” beta and gamma rhythms activity known to be involved in cognitive processes (Zelano et al., 2016). Data demonstrating the potential role of RRO in cognitive processing has accumulated in recent years also from human studies (Zelano et al., 2016; Arshamian et al., 2018; Perl et al., 2019). In humans, behaviors modulated by respiratory phase include eye (Rittweger and Popel, 1998; Rassler and Raabe, 2003) and finger (Ebert et al., 2002; Nassrallah et al., 2013) movements, visual (Li et al., 2012)and auditory (Gallego et al., 1991)reaction times, grip-force (Li and Laskin, 2006), olfactory memory consolidation (Arshamian et al., 2018), aversive associative learning (Waselius et al., 2019), visuospatial cognition (Perl et al., 2019), and visual working memory retrieval (Nakamura et al., 2018).

The strategic use of brain oscillations as a mesoscale mechanistic link between cellular and circuit-level neurophysiology and brain-wide network activity giving rise to cognition and behavior has borne fruit in research on schizophrenia (Siok et al., 2006; Ford et al., 2007; Hajos et al., 2008; Lanre-Amos and Kocsis, 2010; Kocsis, 2012; Driesen et al., 2013; Harvey et al., 2013; Kocsis et al., 2013, 2014; Khlestova et al., 2016; Pittman-Polletta et al., 2018; Parker et al., 2019; Hamilton et al., 2020; Thorn et al., 2022), Parkinson's disease (Brown, 2003; Oswal et al., 2013; Little and Brown, 2014; Li and Zhang, 2015; Johnson et al., 2021), and many other pathological conditions, e.g., epilepsy (Buzsaki et al., 1990; Steriade, 2005; Beenhakker and Huguenard, 2009; Takeuchi and Berenyi, 2020), autism (Ben-Ari, 2015; Casanova et al., 2020; Kayarian et al., 2020; Jia et al., 2021), dyslexia (Hancock et al., 2017; Vidyasagar, 2019), and neurodegeneration (Rossini et al., 2007; Nimmrich et al., 2015). The functions, dynamics, and key features including characteristic frequencies of brain oscillations are similar in humans and rodents, and they have been shown to be not only robust but heritable (van Pelt et al., 2012) and responsive to interventions, making them a valuable tool for translational research.

**Figure 1:**
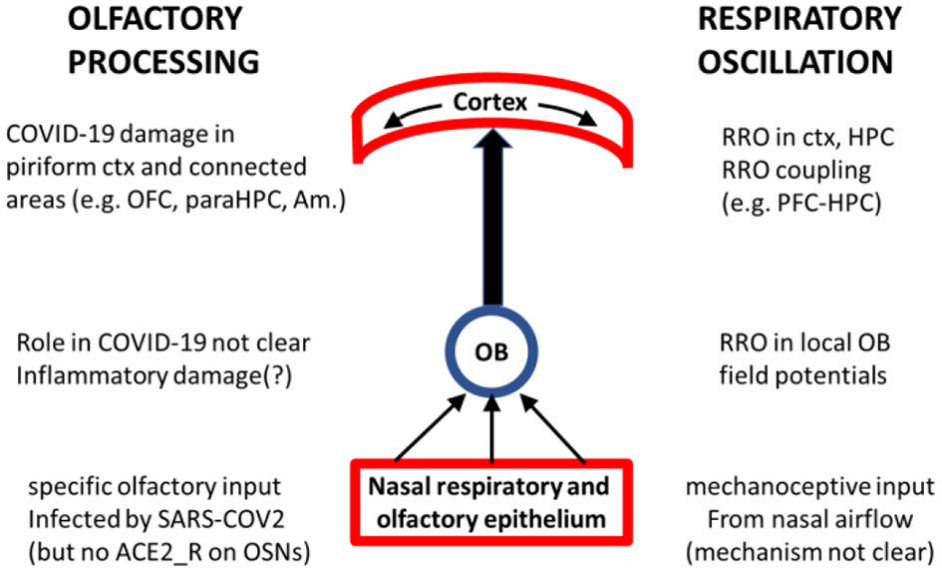
Schematics of key nodes of the olfactory processing system **(middle)** with their involvement in COVID-19 **(left)** and in RRO **(right)**. Olfactory and respiratory epithelium plays crucial role in generating both signals and is the primary site of SARS-CoV-2 infection. OB is transmitting ascending olfactory sensation as well as mechanoceptive signal of rhythmic nasal airflow and is receiving “top-down” information related to olfactory processing (from ctx, HPC, Amygdala) as well as oscillatory drive (e.g. theta, gamma from HPC); reports of its possible COVID-19 inflammatory damage are not fully consistent (Sherif et al., 2022; Abdou et al., 2023; Muccioli et al., 2023). Piriform cortex is the primary target of the olfactory tract where cortical processing of olfactory information involves RRO-driven local gamma oscillations (Gonzalez et al., 2023) and is transmitted to other cortical structures, oscillatory coupled at different frequencies [including RRO (Mofleh and Kocsis, 2021a)], for further processing in the context of different cognitive functions. COVID-19 impairment of these cortical structures is well-documented.

We believe that RRO may provide mechanistic insight into both ends of COVID-19 related olfactory dysfunction, shedding light on both questions posed above (see Figure 1) and may be important for investigations of olfactory processing in general and its COVID-19 related pathology, in particular. RRO adheres to the principle of hierarchical organization of brain oscillations in which slow rhythms (delta, theta, alpha, etc.) modulate local gamma oscillations to facilitate functional coupling of local and distant networks. Gamma is present in all cortical networks and in the OB (Beshel et al., 2007; Brea et al., 2009), as well. RRO couples with slow rhythms intrinsically generated in cortical networks (Kocsis et al., 2018; Mofleh and Kocsis, 2021a) and modulates cortical gamma (Cavelli et al., 2020; Gonzalez et al., 2023). It was recently shown that RRO-gamma coupling in the piriform cortex acted to select and amplify the best set of neurons for representing the odor sensed during a sniff, and to quieten less relevant neurons (Gonzalez et al., 2023), pointing to the strong involvement of RRO in olfactory processing at every level of organization from the OB to higher structures (Figure 1). Thus, our hypothesis concerning RRO does not suggest a separate channel to COVID-19 pathology, alternative to olfactory disfunction. It may rather suggest that considering RRO may provide a significant contribution to investigations of the neuropsychiatric sequelae of olfactory dysfunction related to COVID-19 infection. This latter is rapidly progressing, extending rigorously designed longitudinal MRI studies (Douaud et al., 2022) to comparing COVID-19 patients with or without olfactory dysfunction (Delgado-Alonso et al., 2022; Yus et al., 2022; Caroli et al., 2023) and alterations in functional connections between parahippocampal gyrus and orbitofrontal cortex or other brain regions associated with sensory processing and cognitive functioning in groups of healthy controls, vs. COVID-19 with vs. without smell loss (Díez-Cirarda et al., 2022; Wingrove et al., 2023). Below, we describe the potential links between RRO and cognitive function and dysfunction, and between olfactory dysfunction and impaired RRO (about which less is known), in greater detail.

## Non-olfactory RRO input from OB is strongly involved in cortical processing and cognitive function

Large potential waves in OB and piriform cortex rhythmically occurring at each inspiration have been demonstrated over 80 years ago (Adrian, 1942) and adjustment of the respiratory rate to the frequency of HPC theta rhythm, invariably present during stereotyped sniffing bouts, was reported several decades later (Macrides, 1975; Macrides et al., 1982; Semba and Komisaruk, 1984). These findings initiated highly productive research clarifying the cellular mechanisms involved, how they are adapted to different behaviors and cognitive tasks, and how they are affected by numerous pharmacological compounds [rev. (Klemm, 1976; Kepecs et al., 2006; Kay et al., 2009; Kay, 2014; Tort et al., 2018a; Heck et al., 2019)]. As a result, the vital engagement of HPC in olfactory processing is well established. Theta rhythm generated in HPC controls multiple processes in the olfactory system from the OB (Kepecs et al., 2006; Rojas-Libano et al., 2018; Liu et al., 2020) to the piriform cortex in both rodents (Wilson et al., 2011; Xu and Wilson, 2012; Morrison et al., 2013; Kay, 2014; Trieu et al., 2015; Dupin et al., 2020; Iravani et al., 2021; Sheriff et al., 2021; Poo et al., 2022) and human (Jiang et al., 2017; Iravani et al., 2021; Yang et al., 2022). Theta rhythm from HPC also synchronizes the olfactory system with multiple non-olfactory sensory channels and associated motor control of rhythmic nasal, whisker, and head movements to further optimize odor perception. Thus, theta rhythm synchronized with RRO occupies a central position in a complex system considered a “paradigmatic example” of active sensing (Wachowiak, 2011; Corcoran et al., 2018) aimed at processing synchronized streams of olfactory and other (e.g. tactile, visual, etc.) information.

More recently, an explosion of findings firmly demonstrated that brain activity and cognitive function are also modulated by respiratory rhythm outside of sniffing episodes, as well [rev. (Tort et al., 2018a; Heck et al., 2019)]. Slow, non-theta RRO were detected in numerous brain structures, including higher order cognitive centers as the prefrontal cortex (Biskamp et al., 2017; Zhong et al., 2017) and HPC (Yanovsky et al., 2014; Chi et al., 2016; Lockmann et al., 2016). RRO coupling with wide-spread forebrain activity was confirmed using advanced techniques, including single unit firing (Rojas-Libano and Kay, 2008; Chi et al., 2016; Biskamp et al., 2017; Zhong et al., 2017; Koszeghy et al., 2018; Jung et al., 2022, 2023), current source density (Rojas-Libano and Kay, 2008; Chi et al., 2016; Lockmann et al., 2016), and phase modulation of local gamma activity (Ito et al., 2014; Biskamp et al., 2017; Zhong et al., 2017; Rojas-Libano et al., 2018; Cavelli et al., 2020). It was firmly established that RRO derives from rhythmic nasal airflow in the OB (Yanovsky et al., 2014), which dynamically couples with intrinsic network oscillations in higher brain structures (Kocsis et al., 2018) either: (1) by coherence, when the frequency of RRO matches that of local field potentials such as delta and theta activity in rodents (Ito et al., 2014; Yanovsky et al., 2014; Chi et al., 2016; Lockmann et al., 2016; Biskamp et al., 2017; Tort et al., 2018b), or (2) by phase-amplitude modulation when the frequencies diverge, as in gamma high frequency oscillations (HFO; >100 Hz) in rodents (Ito et al., 2014; Yanovsky et al., 2014; Biskamp et al., 2017; Zhong et al., 2017) or all characteristic EEG rhythms in human (which have frequencies comparable to those in rodents, but faster than human respiration) (Zelano et al., 2016).

Importantly, the effect of RRO driven by mechanoceptive input from the OB goes well beyond rhythmic modulation of the level of activity in higher brain structures; it is deeply involved in complex circuit mechanisms of neural network function. This is an area of intense on-going investigations on different levels of network organization, from cellular to interregional communication (Rojas-Libano and Kay, 2008; Ito et al., 2014; Chi et al., 2016; Lockmann et al., 2016; Biskamp et al., 2017; Zhong et al., 2017; Koszeghy et al., 2018; Rojas-Libano et al., 2018; Cavelli et al., 2020; Mofleh and Kocsis, 2021a; Jung et al., 2022, 2023; Gonzalez et al., 2023). Although OB projection to different higher brain regions is not direct (Hoover and Vertes, 2007; Mori et al., 2013; Yanovsky et al., 2014; Moberly et al., 2018), mostly mediated by the piriform cortex, RRO appears in functionally different areas dynamically coupled in a complex behavior- and task-related manner. As RRO depends on vigilance state (Girin et al., 2020; Mofleh and Kocsis, 2021a), it appears coincident with various state-dependent intrinsic brain oscillations which exhibit characteristic spatial distributions. For example, transient time windows of long-range cortico-cortical coupling of gamma activity (a phenomenon implicated in visual perception, attention, and bottom-up information transfer) are regularly evoked at a specific time during each breathing cycle, as high frequency oscillations e.g. in the frontal cortex are phase-coupled with OB and consequently with piriform cortex (González et al., 2023). Frontal cortex and HPC, typically generating delta and theta oscillations, respectively, are accessible for rhythmic OB input depending on the behavior-dependent respiratory rate, and this has strong implications for their communication. We have shown recently that in resting states, slow (~2 Hz) respiration firmly couples with frontal cortex providing a delta communication channel toward HPC with weaker and variable RRO (Mofleh and Kocsis, 2021a,b)—i.e. in contrast with the well known dominant theta-driven communication controlled by HPC during exploration. In association areas, e.g. in parietal cortex (recorded far caudal from primary olfactory areas) where RRO and intrinsic brain oscillations are driven by converging extrinsic inputs transmitted from different sources, the two rhythms may simultaneously activate partially overlapping cellular populations at different strengths depending on vigilant states, even though the laminar profiles of theta and RRO diploes (a different level of organization) are in different layers (Jung et al., 2022, 2023).

## COVID-19 mechanisms in the olfactory epithelium, affecting smell and possibly RRO

Potential pathomechanisms of COVID-19 related olfactory dysfunction have been extensively studied and regularly reviewed in the past several years. At the very beginning (Summer of 2020) for example, Cooper et al. (2020) pointed out, that the natural history of COVID-19-associated anosmia argues that SARS-CoV-2 attacks the olfactory system through mechanisms distinct from those used by the more benign endemic coronaviruses (Giacomelli et al., 2020; Spinato et al., 2020). In fact, imaging studies of the olfactory bulb in COVID-19 patients were either normal or revealed focal inflammation (Eliezer et al., 2020). According to current understanding, SARS-CoV-2 does not directly infect OSNs; COVID-19 induced OSN dysfunction is mediated instead by alterations to the microenvironment maintained by angiotensin converting enzyme-2 (ACE2) receptor-expressing cells in the olfactory epithelium [rev. (Cooper et al., 2020; Iadecola et al., 2020; Las Casas Lima et al., 2022; Rodriguez-Sevilla et al., 2022; Butowt et al., 2023)]. It is believed that the primary target of SARS-CoV-2 infection in the olfactory mucosa are sustentacular cells, known to express ACE2 receptors (Bilinska and Butowt, 2020; Bilinska et al., 2020; Brann et al., 2020; Fodoulian et al., 2020; Klingenstein et al., 2020; Ye et al., 2021; Shahbaz et al., 2022). Moreover, the virus was demonstrated directly in these cells while still replicating in COVID-19 patients who died a few days after infection (Khan et al., 2021).

Rodent sustentacular cells have been ascribed myriad roles collectively referred to as “supporting”: absorptive, detoxifying, metabolic, nourishing, phagocytic, physical, secretory, structural (Getchell et al., 1989; Hansel et al., 2001; Kam et al., 2014; Liang, 2020; Butowt and von Bartheld, 2021; Khan et al., 2021). The tight anatomical and functional links between OSNs and sustentacular cells is a strong indication that impairment of the latter would affect the function of the former. Through changes in extracellular ionic concentrations, nutrient bioavailability, or structural support, alterations in the microenvironment of OSNs could easily cause disruptions in both the perception of odorants and the synchronization of brain-wide electrical activity. The cellular mechanisms of RRO generation in the OB are not yet clear at this level of detail, and we are not aware of published research on whether impaired RRO are associated with COVID-19 pathology. But it seems plausible that impaired function of non-sensory olfactory epithelial cells may negatively affect RRO. Indeed, the potential for metabolic factors to instigate changes in brain rhythms has been demonstrated, e.g., in recent biophysical models of burst suppression under propofol anesthesia (Ching et al., 2012) and the sleep-stage architecture of thalamocortical spindles (Roberts, 2007).

The causal relationship between OSN dysfunction and potential dysrhythmias is not clear *a priori*. Disruption of OSN function might directly lead to impaired RRO, as OSNs can respond not only to odorants but also to mechanical stimuli (Connelly et al., 2015; Grosmaitre et al., 2021) and transmit both odor and air flow-driven mechanical signals (Carey et al., 2009; Iwata et al., 2017). Such mechanosensory activity has been extensively studied as a mechanism in sniffing-related synchronization of brain activity. However, its role in the generation of lower (i.e., delta) frequency RRO targeting a wider range of forebrain regions remains unidentified. In the opposite direction, mechano-sensation of rhythmic airflow and its deficits (i.e., the disorganization of RRO) may directly affect odor perception already at the level of OB. Odor encoding occurs relative to the phase of respiration (Kepecs et al., 2006; Cury and Uchida, 2010), i.e. during inhalation, and its frequency determines many aspects of OB activity (David et al., 2015; Short et al., 2016). Olfactory external tufted cells exhibit rhythmic bursting activity in several frequency ranges synchronized within olfactory glomeruli (Hayar et al., 2004; De Saint Jan et al., 2009) by multiple mechanisms including gap junction connectivity, slow (dendritic) excitatory currents, and slow recurrent inhibition from periglomerular cells (Hayar et al., 2005), and most likely mediate the phase-locking of OB output to respiration (Buonviso et al., 2003). Thus, impaired RRO may directly contribute to COVID-19 associated olfactory deficits.

## Discussion

In this opinion paper we advocated for the investigation of potentially impaired non-olfactory inputs arising from the olfactory epithelium and involved in cognitive function (e.g., RRO) as a potential mechanistic factor underlying the neuropsychiatric consequences of SARS-CoV-2 infection and linking them to COVID-19 related olfactory dysfunction. We should mention however, that long-Covid is a new and very complex condition, which includes many mechanisms—viral, inflammation, signaling pathways and, of course, non-homogeneous, depending on the acute phase, virus & patients particularities. RRO is one potential component in this puzzle that should be considered.

Additionally, given the well established links between olfactory function and mental health, RRO are likely to play a significant role in other medical conditions (MacKay et al., 2018) as well, when these oscillations may be disrupted for different reasons, or when this extrinsic synchronizing input remain functional while intrinsic brain oscillations are disturbed. An obvious example of the first, besides impairments of the olfactory epithelium, is the condition of long-term intubation, necessary in the context of a variety of medical indication and treatment. Its potential consequences on cognition are hard to distinguish from those directly related to the basic pathology. However, promoting RRO in sensory and motor cortex through the activation of oro-facial and neck muscle activity in synchrony with respiration (Wachowiak, 2011; Corcoran et al., 2018) may have therapeutic benefits for both weaning procedures prior to extubation, and subsequent rehab. As for the second possibility, abnormal brain oscillations, “oscillopathies”, are commonly found in a wide variety of psychiatric diseases associated with severe cognitive deficits (see e.g. Katsuki et al., 2022; Shu et al., 2022; Sohal, 2022; Syed et al., 2022; Beste et al., 2023; Ramos et al., 2023; Wischnewski et al., 2023 for recent reviews). Rhythmic nasal airflow continues uninterrupted, but the potential alterations to the functionality of cortical RRO remain unclear. We have shown recently for example that normal RRO patterns (Mofleh and Kocsis, 2021a,b) remain functional after severe disruption of intrinsic cortical and HPC oscillations under the psychotomimetic Ketamine, in a state characterized by “psychotic-like” behavior and abnormal cortical gamma activity, even with a highly unstable respiratory rate (Staszelis et al., 2022). Whether and to what extent this source of extrinsic oscillatory drive provides a mechanism for interregional long-range oscillatory coupling between cortical networks requires further investigations in specific disorders.

## Author contributions

All authors listed have made a substantial, direct, and intellectual contribution to the work and approved it for publication.
